# Detection, isolation and characterization of phage-host complexes using BONCAT and click chemistry

**DOI:** 10.3389/fmicb.2024.1434301

**Published:** 2024-09-03

**Authors:** Patrick Hellwig, Anna Dittrich, Robert Heyer, Udo Reichl, Dirk Benndorf

**Affiliations:** ^1^Chair of Bioprocess Engineering, Otto-von-Guericke University Magdeburg, Magdeburg, Germany; ^2^Bioprocess Engineering, Max Planck Institute for Dynamics of Complex Technical Systems Magdeburg, Magdeburg, Germany; ^3^Department of Systems Biology, Institute of Biology, Otto-von-Guericke University Magdeburg, Magdeburg, Germany; ^4^Multidimensional Omics Analyses Group, Leibniz-Institut für Analytische Wissenschaften – ISAS – e.V., Dortmund, Germany; ^5^Multidimensional Omics Analyses Group, Faculty of Technology, Bielefeld University, Universitätsstraße, Bielefeld, Germany; ^6^Department of Microbiology, Anhalt University of Applied Sciences, Köthen, Germany

**Keywords:** BONCAT, click chemistry, bacteriophage, biotin, proteomics, LC–MS/MS, host screening, fluorescence

## Abstract

**Introduction:**

Phages are viruses that infect prokaryotes and can shape microbial communities by lysis, thus offering applications in various fields. However, challenges exist in sampling, isolation and accurate prediction of the host specificity of phages as well as in the identification of newly replicated virions in response to environmental challenges.

**Methods:**

A new workflow using biorthogonal non-canonical amino acid tagging (BONCAT) and click chemistry (CC) allowed the combined analysis of phages and their hosts, the identification of newly replicated virions, and the specific tagging of phages with biotin for affinity chromatography.

**Results:**

Replication of phage λ in *Escherichia coli* was selected as a model for workflow development. Specific labeling of phage λ proteins with the non-canonical amino acid 4-azido-L-homoalanine (AHA) during phage development in *E. coli* was confirmed by LC–MS/MS. Subsequent tagging of AHA with fluorescent dyes via CC allowed the visualization of phages adsorbed to the cell surface by fluorescence microscopy. Flow cytometry enabled the automated detection of these fluorescent phage-host complexes. Alternatively, AHA-labeled phages were tagged with biotin for purification by affinity chromatography. Despite biotinylation the tagged phages could be purified and were infectious after purification.

**Discussion:**

Applying this approach to environmental samples would enable host screening without cultivation. A flexible and powerful workflow for the detection and enrichment of phages and their hosts in pure cultures has been established. The developed method lays the groundwork for future workflows that could enable the isolation of phage-host complexes from diverse complex microbial communities using fluorescence-activated cell sorting or biotin purification. The ability to expand and customize the workflow through the growing range of compounds for CC offers the potential to develop a versatile toolbox in phage research. This work provides a starting point for these further studies by providing a comprehensive standard operating procedure.

## Introduction

Phages are viruses that infect prokaryotes and play a major role in the composition and evolution of microbial communities (Anderson et al., 2011; Heyer et al., 2019b; Howard-Varona et al., 2017; Kristensen et al., 2010; Marsh and Wellington, 1994; Suttle, 2007). Phages are also considered a potential alternative to antibiotics for infection control (Clark and March, 2006; Fernández et al., 2021; Kutateladze and Adamia, 2010; Sieiro et al., 2020). Therefore, the identification and characterization of phages in natural, biotechnological and clinical areas, including patients, is of considerable interest.

So far, DNA sequencing and genome annotation are crucial for phage detection and characterization. However, associating an annotated phage DNA sequence with a corresponding host is challenging (Hwang et al., 2023). Furthermore, DNA sequencing does not cover RNA phages (Fernández et al., 2021; Gregory et al., 2019; Paez-Espino et al., 2016) and cannot distinguish whether a phage genome is expressed or only integrated into the genome of the host cell. Heyer et al., 2019b demonstrated that metaproteomics helps to close these knowledge gaps by analyzing the expression of phage proteins in microbial communities. Nevertheless, analyzing phages in complex microbial communities remains challenging due to their low contribution to the total biomass, the elaborate methods required for phage enrichment and the lack of approaches to co-enrich their hosts.

The enrichment and purification of low-abundant phages and the visualization of phage-host complexes are keys to overcome these limitations. In the past, developments have been made to detect phages together with their hosts by staining the phage genome and by isolating phages attached to their host (Deng et al., 2014; Džunková et al., 2019; Ohno et al., 2012). There have also been attempts to analyze phages with their host at the single cell level (Sakowski et al., 2021). These methods efficiently allow to detect phages and hosts and are used for the sequencing of phage genomes in conjunction with the host genome, making cultivation unnecessary in many cases. However, they often need prior knowledge of phage genomes for fluorescent dye selection and prone to non-specific staining (Low et al., 2020). In general, the adsorption of the phage does not inevitably result in infection (Džunková et al., 2019).

The tagging of phage proteins represents a novel innovative alternative approach that allows the specific identification of newly replicated phages independent of the genome (RNA/DNA) while recognizing important proteins involved in the host infection process. Labeling of proteins, particularly by biorthogonal non-canonical amino acid tagging (BONCAT) and click chemistry (CC), represents a versatile approach to mark phage proteins (Dieterich et al., 2006; Kolb et al., 2001). CC is very flexible as for example, fluorescent dyes of a wide range of wavelengths and affinity markers with different properties can be attached to the proteins, which makes this technology easily adaptable to the existing laboratory equipment.

The bottleneck of this technology is the necessity to cultivate phages and hosts for effective labeling, and it is crucial that the phages are adequately labeled with ncAAs. Hatzenpichler et al. (2014) demonstrated the efficacy of labeling newly synthesized proteins in microbial communities using BONCAT and CC. Pasulka et al. (2018) further extended this approach, utilizing fluorescence microscopy to quantify virulent phage replication. Additionally, a new labeling method called “THRONCAT” shows promise in addressing the issues of insufficient labeling effectiveness in the future (Ignacio et al., 2023).

**Figure 1:**
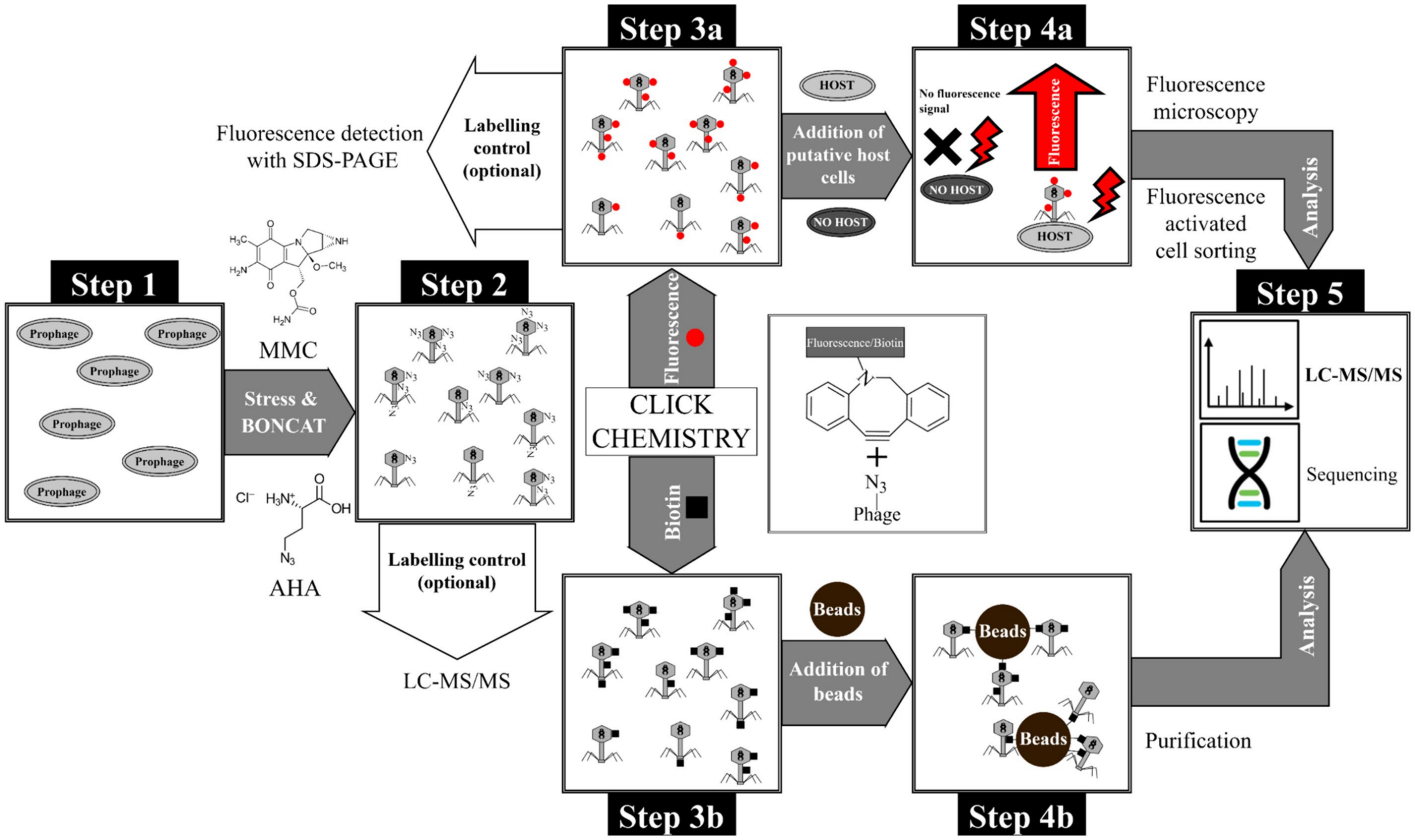
Overview of the BONCAT workflow for detection of phages and purification of phage-host complexes. Step 1: Cultivation of *E. coli* with genome-integrated phage λ, Step 2: Induction of phage replication with MMC and subsequent incorporation of the non-canonical amino acid AHA into phage proteins. The incorporation of AHA into newly synthesized proteins was subsequently verified by LC–MS/MS. Step 3: Tagging of AHA-labeled phages with (a) fluorophores or (b) biotin using CC. In-gel detection is possible using a fluorescent dye (after CC) as quality control for CC. Step 4a: Incubation of fluorescent phages with putative host cells and identification/sorting of adsorbed phages by fluorescence microscopy and flow cytometry, respectively. Step 4b: Purification of biotin-labeled phages using magnetic beads (monomeric avidin beads). Step 5: Analysis of purified phages or phage-host complexes by LC–MS/MS or sequencing (not used in this study).

In this paper we demonstrate successful isolation of phage host complexes from a laboratory culture system and thus provide a basis for possible future applications in microbial ecology. In particular, we present a workflow (Figure 1) that allows to enrich and analyze phage-host complexes. The BONCAT workflow depends on incorporating 4-azido-L-homoalanine (AHA) into newly synthesized phage proteins. Combined with copper-free CC, fluorescent dyes or biotin were attached to AHA-labeled phages without denaturation. Combining BONCAT and CC allowed the detection of phage proteins adsorbed to the host surface and the specific enrichment of labeled phages. The workflow was evaluated using *E. coli* and phage λ as a well-established model system.

### Materials and methods

The workflow established is shown in Figure 1. A detailed description of the methods, including a step-by-step standard operation procedure, can be found in Supplementary material S2. Briefly, replication of phage λ was induced with mitomycin C (MMC) and the newly synthesized phage λ proteins were labeled with AHA (Figure 1, step 1 and 2). After centrifugation and filtration of the AHA-labeled phages, CC was used to attach fluorescent dyes or biotin (Figure 1, step 3a and b). Coupling of fluorescent dyes to phages enabled the detection of phages adsorbed to their host cells by fluorescence microscopy and flow cytometry (Figure 1, step 4a). In addition, biotin coupling allowed the purification and enrichment of phages via affinity chromatography (Figure 1, step 4b). This enrichment allowed the detection of the phages by liquid chromatography-mass spectrometry/mass spectrometry (LC–MS/MS). Alternatively, well established approaches for DNA/RNA sequencing could be applied at this step.

#### Induction and replication of AHA-labeled phages

**Figure 2:**
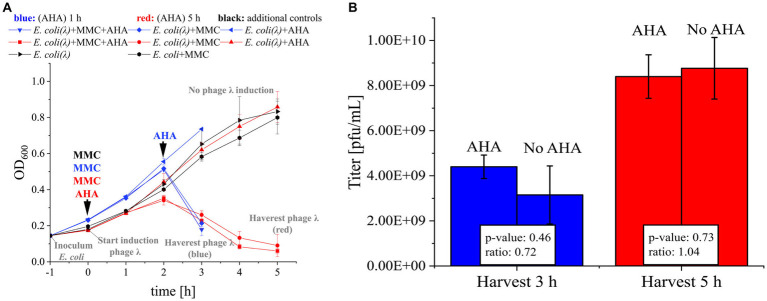
MMC induction and AHA labeling of phage λ. **(A)** *E. coli* (DSM 5911, control for toxicity of MMC) and *E. coli* with integrated phage λ (*E. coli* (λ)) were cultivated. The time course of OD_600_ for 3 h (blue) resp. 5 h (red) was recorded. MMC was added to the indicated cultures after 1 h of incubation (0 h). Corresponding cultures were marked with “+ MMC.” AHA was added (“+ AHA”) at the same time as MMC (AHA 5 h, red) or 1 h before the drop of the OD_600_ (AHA 1 h, blue). In each case, controls were included that were cultivated with MMC only, AHA only, or without either (as indicated)., see Supplementary Protocol S1; **(B)** Phage titer (pfu/mL) determined by plaque assay of the harvested phages from A. Mean and standard deviation of the phage λ titer for three independent experiments; differences in titer not significant (*p* > 0.05; t-test with Benjamini-Hochberg correction). Raw data: see Supplementary Table S1.

*Escherichia coli* K12 (DSM 5911) with genome-integrated phage λ and *E. coli* K12 (DSM 5911) without phage integration were cultured in M9 minimal medium at 37°C and 130 rpm overnight. Overnight cultures diluted to an optical density of 600 nm (OD_600_) ≈ 0.145 were used as inoculum to start new batches. After incubation of the bacteria for 1 h at 37°C and 130 rpm, 0.5 μg/mL MMC and/or 0.1 mM AHA were added as indicated in Figure 2 and Supplementary Table M1. Samples were taken to monitor bacterial growth, and OD_600_ was measured every hour. After 4 h or 6 h, phage λs were harvested by centrifugation of the culture (3,000 × g, 12 min, 4°C). The supernatant was collected, and samples were adjusted to pH 7 with 1 M NaOH. Next, samples were clarified using a syringe with a filter cascade (5 μm, 1.2 μm, 0.8 μm, 0.45 μM, Sartorius AG). The cell pellets were not further analyzed. For details, see Supplementary Protocol S1.

#### Plaque-assay

The phage suspensions were sequentially diluted (10^−6^ to 10^−7^), and 100 μL of each dilution was mixed with 4 mL of melted overlay agar and 100 μL of a fresh overnight culture of *E. coli* K12. Mixtures were poured onto a plate with underlay agar. Hardened agar plates were incubated at 37°C overnight. Based on the number of plaques on plates, the phage titer was calculated: Number of plaques × 10 × reciprocal of dilution = pfu/mL. For details, see Supplementary Protocol S2.

#### Protein extraction, protein quantification, and sample preparation for LC–MS/MS

Proteins from 4 mL of each phage suspension were extracted with chloroform-methanol. Then, proteins were resuspended in 1 mL 8 M urea buffer. The protein concentration was quantified using amido black assay. 25 μg of total protein was used for Filter Aided Sample Preparation (FASP) digestion with MS-approved trypsin (1:100 μg protein) (Heyer et al., 2019a). The resulting peptide solutions were dried in a vacuum centrifuge and solubilized in 75 μL loading buffer A [LC–MS water and 0.1% trifluoroacetic acid (TFA)]. For details, see Supplementary Protocol S3.

#### LC–MS/MS

LC–MS/MS analysis was performed using an UltiMate® 3,000 nano splitless reversed-phase nanoHPLC (Thermo Fisher Scientific, Dreieich) coupled online to a timsTOF™ pro mass spectrometer (Bruker Daltonik GmbH, Bremen). For details, see Supplementary Protocol S3.

#### Identification of AHA incorporation using MASCOT

MS/MS raw data files were processed with the Compass DataAnalysis software (version 5.3.0, Bruker Corporation, Bremen, Germany) and converted to Mascot Generic Files (.mgf). The files were uploaded to MASCOT Daemon (Version 2.6.0) (Perkins et al., 1999) and searched against a filtered UniProt database containing only *E. coli* K12 (taxonomy_id: 83333, 23.03.2023) and *Enterobacteria phage lambda* (taxonomy_id: 10710, 23.03.2023) entities. The following modifications were used: oxidation of methionine, carbamidomethyl, AHA, and reduced AHA (see Supplementary Table M2).

#### Fluorophore/biotin tagging of BONCAT phages by click chemistry

Phage suspensions were collected on a 100 kDa filter via centrifugation (5 min, 3,500 × g, RT). Afterwards, phosphate-buffered saline (PBS) was added and samples were centrifuged again (5 min, 3,500 × g, RT). 100 mM iodoacetamide in PBS was added, and samples were incubated in the dark at 37°C for 1 h. Afterwards, 0.15 μM dibenzylcyclooctyne (DBCO)-cyanin 5.5 or 0.15 μM DBCO-Alexafluor 555 or 0.15 mM DBCO-PEG_4_-biotin were added. Next, samples were incubated in the dark for 30 min at 37°C, washed thrice with PBS, and resuspended in 1 mL PBS. Finally, phage suspensions were transferred to 1.5 mL LoBind® tubes and stored at 4°C in the dark. For details, see Supplementary Protocol S4.

#### Specific adsorption of labeled phages to host cells

*Escherichia coli* and *Pseudomonas fluorescens* (DSM 50090) were cultured in standard nutrient broth (Carl Roth, # 1533.1, plus 5 mM MgSO_4_) at 37°C, 130 rpm overnight. Next, bacteria were diluted with fresh standard nutrient broth in sterile 1.5 mL tubes to 1.80 × 10^7^ cells/mL and incubated for 20 min at 30°C and 600 rpm. Fluorescent phages were added to adjust a multicity of infection (moi) ≈ 2. As a control, only medium was added to the bacteria. 200 μL samples were taken after 0 min, 10 min, 20 min, 30 min, and 60 min. The samples were immediately centrifuged (5 min, 16,400 × g, 4°C). The supernatants of the samples were removed, and cell pellets were immediately fixed with 4% formaldehyde in PBS for 1 h at 4°C. The fixation solution was removed by centrifugation (5 min, 16,400 × g, 4°C). Cells were resuspended in PBS and stored at 4°C. For details, see Supplementary Protocol S4.

#### Fluorescence microscopy

Phage-host complexes were visualized with an Imager.M1 fluorescence microscope (Carl Zeiss, Jena, Germany) using a 100X objective (EC-Neoflur 100x/1.3 Oil Ph3) and phase contrast. For details, see Supplementary Protocol S4.

#### Flow cytometry

Flow cytometric analysis was performed using a FACS Canto II equipped with three lasers (405 nm, 488 nm, 663 nm), Firmware Version 1.47 (BD Biosciences, Franklin Lakes, NJ, United States). The data were analyzed with the software FlowJo™ (BD Biosciences,10.8.1). For details, see Supplementary Protocol S4.

#### Native purification of biotinylated phages via magnetic beads

Biotinylated phages were purified with BcMag™ Monomeric Avidin Magnetic Beads (Bioclone, MMI-101) kit according to the manufacturer’s instructions. After binding of the phages, beads were washed with PBS. The supernatant of each washing step was collected for further analysis (fraction “Washing phase”). The biotinylated phages bound to the beads were eluted with 2 mM biotin and collected in a new tube (fraction “Elution”). Lastly, the beads were boiled at 60°C for 5 min with an SDS-buffer, and the supernatant was collected for further analysis (fraction “SDS-boiled”). All fractions were analyzed with an untreated control (fraction “Not purified”) with a SDS-PAGE. The phage titer in the “Elution” was also determined with a plaque assay. For details, see Supplementary Protocol S4.

#### SDS-PAGE

SDS-PAGE was performed with 1 mm SDS-PAGE gels with 12% separation and 4% stacking gel (Laemmli, 1970). For details, see Supplementary Protocol S4.

#### Staining and scanning of SDS gels loaded with biotinylated or fluorescent proteins

After electrophoresis and fixation, gels with fluorescent proteins were scanned with Licor Odyssey ODY-2600 (LI-COR Biosciences - GmbH) or Typhoon Trio Variable Mode Imager System (GE Healthcare). Subsequently, the gels were counterstained with Coomassie staining solution overnight and scanned with a Biostep ViewPix900 scanner (Seiko Epson Corporation) (Supplementary Table M3). Gels with biotinylated proteins were fixed, stained with Coomassie, and scanned with a Biostep ViewPix900 scanner (Seiko Epson Corporation) (Supplementary Table M3).

#### In-gel digestion

The method was performed as described in Heyer, Schallert and Büdel et al., 2019a. For each protein band isolated from an SDS gel, 1 μg of protein content was assumed to calculate the amount of MS-approved trypsin (1 μg trypsin:100 μg protein).

#### Replicates, biostatistics, and visualization

All experiments were performed in biological triplicates or as indicated. R-Statistics (version 4.1.2) with R studio (version 2021.09.1 Build 372) was used for statistical analysis. Normal distribution was confirmed by the Shapiro–Wilk test; for group-wise differences, a t-test with Benjamini-Hochberg correction was used.

## Results and discussion

### BONCAT labeling of phage λ in *Escherichia coli*

Efficient phage replication induced by MMC was a critical precondition for the subsequent labeling of phages with AHA. The addition of MMC to exponentially growing *E. coli* resulted in a growth arrest at 2 h and a subsequent decrease of biomass (OD_600_), indicating cell lysis and phage replication (Figure 2A). Based on OD_600_, the addition of AHA for labeling did not reduce phage replication. Similar phage titers confirmed this result for incubations with and without addition of AHA (Figure 2B; *p* > 0.05). Earlier harvest (3 h post infection) resulted in lower phage titers, showing that phage replication was still ongoing until final sampling at 5 h.

In summary, AHA addition did neither inhibit the MMC-induced production of phage λ in *E. coli* nor the production and infectivity of phage λ. This is consistent with recent studies investigating the impact of AHA addition on the growth of *E. coli* (Landor et al., 2023; Steward et al., 2020).

### Verification of the incorporation of AHA by LC–MS/MS

**Figure 3:**
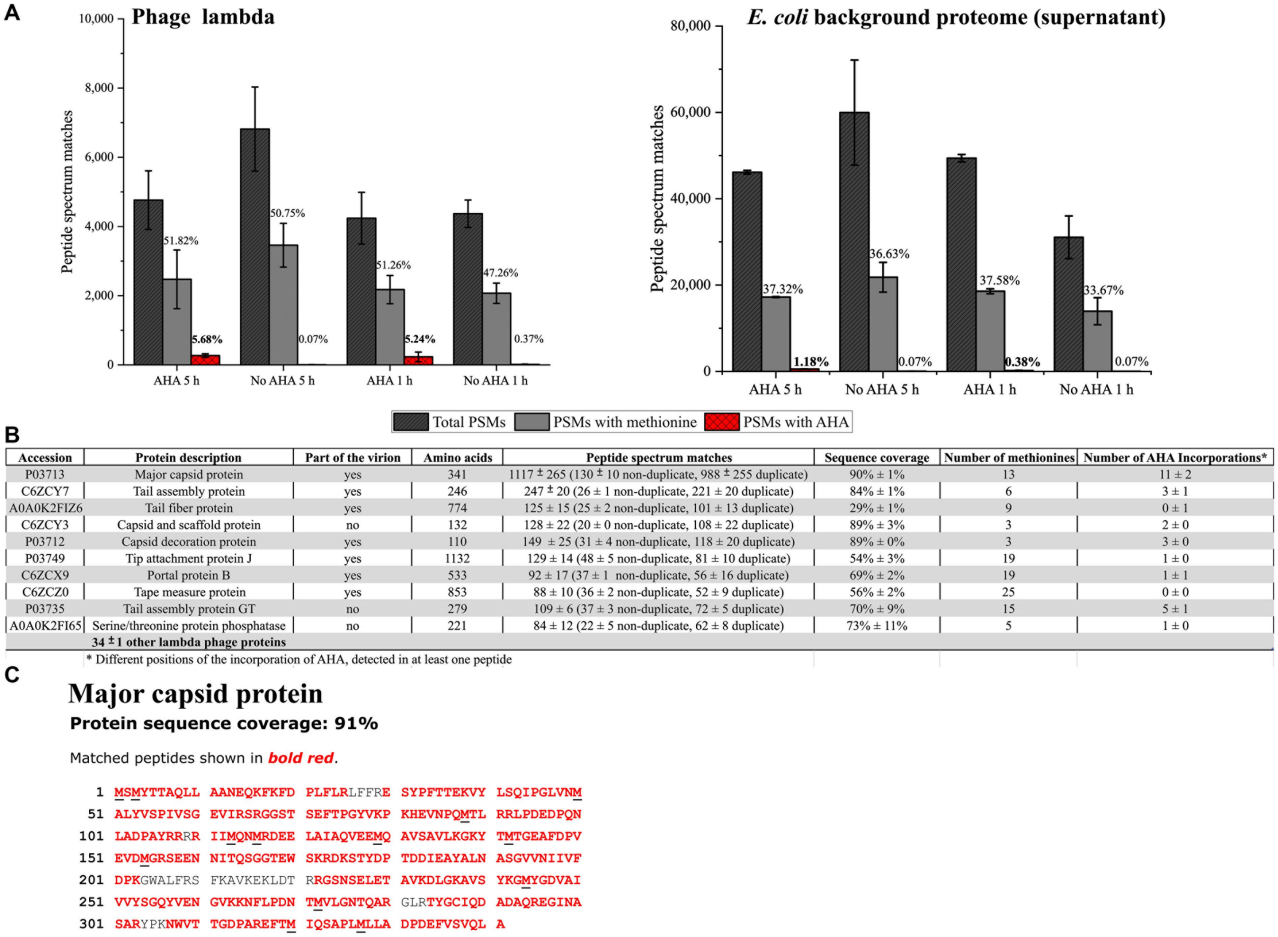
Screening for AHA containing PSMs from phage λ and the *E. coli* background proteome. PSMs were identified by data-dependent acquisition LC–MS/MS using the MASCOT search engine. **(A)** A total number of phage λ and *E. coli* (not cell pellet); only PSMs of the same sample were analyzed. AHA 5 h, AHA 1 h: AHA incubation times of 1 h and 5 h; No AHA 5 h, No AHA 1 h: controls. PSMs with methionine indicate the percentage of methionine-containing PSMs of all identified PSMs. PSMs with AHA indicate the percentage of AHA-containing PSMs of all PSMs. Mean and standard deviation of three independent experiments (Supplementary Table S2). **(B)** The 10 most abundant identified phage λ proteins (by PSM count) containing AHA after MASCOT search for sample AHA 5 h (3 replicates) were analyzed—the order of the 10 phage λ proteins corresponds to replicate 1. PSM: Mean and standard deviation of three replicates. **(C)** The major capsid protein was the most abundant protein detected in all samples (90% ± 1 sequence coverage). Black underlined letters in the amino acid sequence shows the position of methionine.

The successful labeling of proteins with AHA was subsequently confirmed using LC–MS/MS. Here, the incorporation of AHA instead of methionine caused specific mass shifts of tryptic peptides. Overall, LC–MS/MS allowed the assignment of 5,046 ± 1,200 peptide spectrum matches (PSMs) related to phage λ proteins (Figure 3A). After 5 h labeling with AHA, 271 ± 50 PSMs showed incorporation of AHA instead of methionine. Since AHA is incorporated only in place of methionine, the incorporation rate of AHA should be referenced to methionine-containing PSMs (51.82% ± 1.15% of all PSMs). AHA was detected in 5.68% ± 0.23% of all PSMs and in 10.97% ± 0.50% of methionine-containing PSMs. Interestingly, shorter labeling with AHA (1 h) resulted in a similar incorporation rate of AHA (5.24% ± 2.14% of all PSMs) (Figure 3A; Supplementary Table S2), showing that AHA incorporation started soon after addition. Compared to eukaryotic cells, where AHA is only incorporated at 1 out of 400–500 methionine sites (Calve et al., 2016; Kiick et al., 2002; Ngo et al., 2009; van Bergen et al., 2022), the incorporation rate of AHA observed for phage λ was higher. The high incorporation rate of AHA in phage λ proteins has several advantages. A higher incorporation rate provides many reactive sites for subsequent coupling of fluorophores or affinity tags and it may partially compensate for the lower occurrence of methionine in some phages. To further increase the incorporation, the AHA concentration could be further increased (up to 1 mM) or AHA could be added continuously at low concentrations to reduce its impact on the physiology of host cells (Hatzenpichler et al., 2014; Landor et al., 2023; Steward et al., 2020).

Detailed analysis of PSMs allowed to identify 44 ± 1 different phage λ proteins (Figure 3B) associated with the infection cycle. The most abundant phage protein was the major capsid protein, where AHA was incorporated in all methionine positions (Figure 3C). The objective of incorporating AHA should be to provide adequate binding sites for the CC while retaining the functionality of phages. The incorporation of AHA failed only in 1 out of the top 10 identified phage λ proteins (Figure 3B). This result indicates that the high incorporation of AHA may impact the stability or the function of labeled proteins, potentially interfering with the infectivity of the phages (Landor et al., 2023). However, according to the results obtained from plaque assays, labeling with AHA at the given concentration does not significantly affect titers (Figure 2B). Heterogeneity of incorporation of AHA in proteins of phage λ might be caused either by selective incorporation of AHA or by removal of dysfunctional/misfolded proteins after protein synthesis.

During phage-induced cell lysis, the phage harvest may become contaminated with *E. coli* proteins, which could interfere with the subsequent dye or biotin labeling steps of the CC. LC–MS/MS analysis of phage harvest after purification by centrifugation showed the presence of a relatively large number (46,636 ± 11,934 PSMs) of *E. coli* background proteins containing 1.18% ± 0.10% AHA labeled PSMs for 5 h AHA labeling, and 0.38% ± 0.15% AHA labeled PSMs for 1 h AHA labeling. Therefore, shorter labeling with AHA (1 h) in a later phase of infection (2 h after the addition of MMC) should be preferred in situations where labeling of *E. coli* background proteins is detrimental (Figure 3A) despite lower overall labeling efficiency. However, interfering background proteins could also be removed by CsCl centrifugation or PEG precipitation (Boulanger, 2009; Nasukawa et al., 2017; Yamamoto et al., 1970). Nevertheless, every additional purification step might also reduce the yield of phages (Carroll-Portillo et al., 2021).

In summary, both tested AHA incubation periods allowed successful AHA-labeling of the phages. However, the parallel incubation of cells with the phage replication inducer (here MMC) and AHA is more practical, especially for cultures with unknown cell growth dynamics and phage replication kinetics. Therefore, the 5 h incubation period with simultaneous MMC and AHA addition was used in the further course of this study.

### Fluorescence tagging of AHA-labeled phages

AHA-labeling was a precondition for the attachment of fluorescent dyes by CC to identify newly synthesized proteins by SDS-PAGE and fluorescence microscopy (Figure 1 Step 3a) (Dieterich et al., 2007; Hatzenpichler et al., 2014; Pasulka et al., 2018).

Previously published protocols for CC apply precipitation with ethanol to remove excess reagents. However, the denaturation of phages by ethanol precipitation should be omitted for subsequent fluorescence microscopy. Therefore, the protocol for labeling the AHA-labeled phages with fluorophores was adapted so that all CC and washing steps were performed using a 100 kDa filter. The native phages were retained on the filter, while the chemicals passed through in the flow through after CC (Bichet et al., 2021; Bonilla et al., 2016; Erickson, 2009; Hietala et al., 2019). Ultracentrifugation was not considered here, as pelleting phages was considered too time-consuming and potentially reducing overall yield. The newly established filter-based protocol for tagging phages with DBCO Alexafluor (AF) 555 allowed the successful detection of fluorescence in SDS-PAGE (see Supplementary Figure S1). Two fluorescent bands with molecular weights of approximately 37 kDa and 60 kDa were detected for both labeling conditions (1 h and 5 h labeling with AHA). In contrast, no fluorescent proteins were detected in the control sample. The 37 kDa band could correspond to the highly labeled major capsid protein, and the 60 kDa band to the portal protein B. Further, these fluorophore-tagged phages are termed “AF555 phages.” Alternatively, AHA-labeled phages were coupled to DBCO Cyanin (CY) 5.5 by CC (see Supplementary Figure S4). The fluorescence gels showed higher fluorescence intensity with additional bands besides the two main bands at 37 kDa and 60 kDa. In the following, these fluorophore-tagged phages are termed “CY5.5 phages”.

In summary, the AHA-labeled phages had sufficient binding sites for detectable fluorescence tagging via CC. This also confirms the results of the MS measurements, where a high level of incorporation with AHA was found. In addition, sufficient phages could be recovered from the filters for phage protein detection via Coomassie stain and fluorescence detection. This should also allow fluorescence microscopy detection, which will be verified below.

### Detection of phage-host complexes via fluorescence microscopy

The binding of AF555 phages to *E. coli* was confirmed by fluorescence microscopy. AF555 phages were incubated for 30 min with *E. coli* or *P. fluorescens* as negative control.

**Figure 4:**
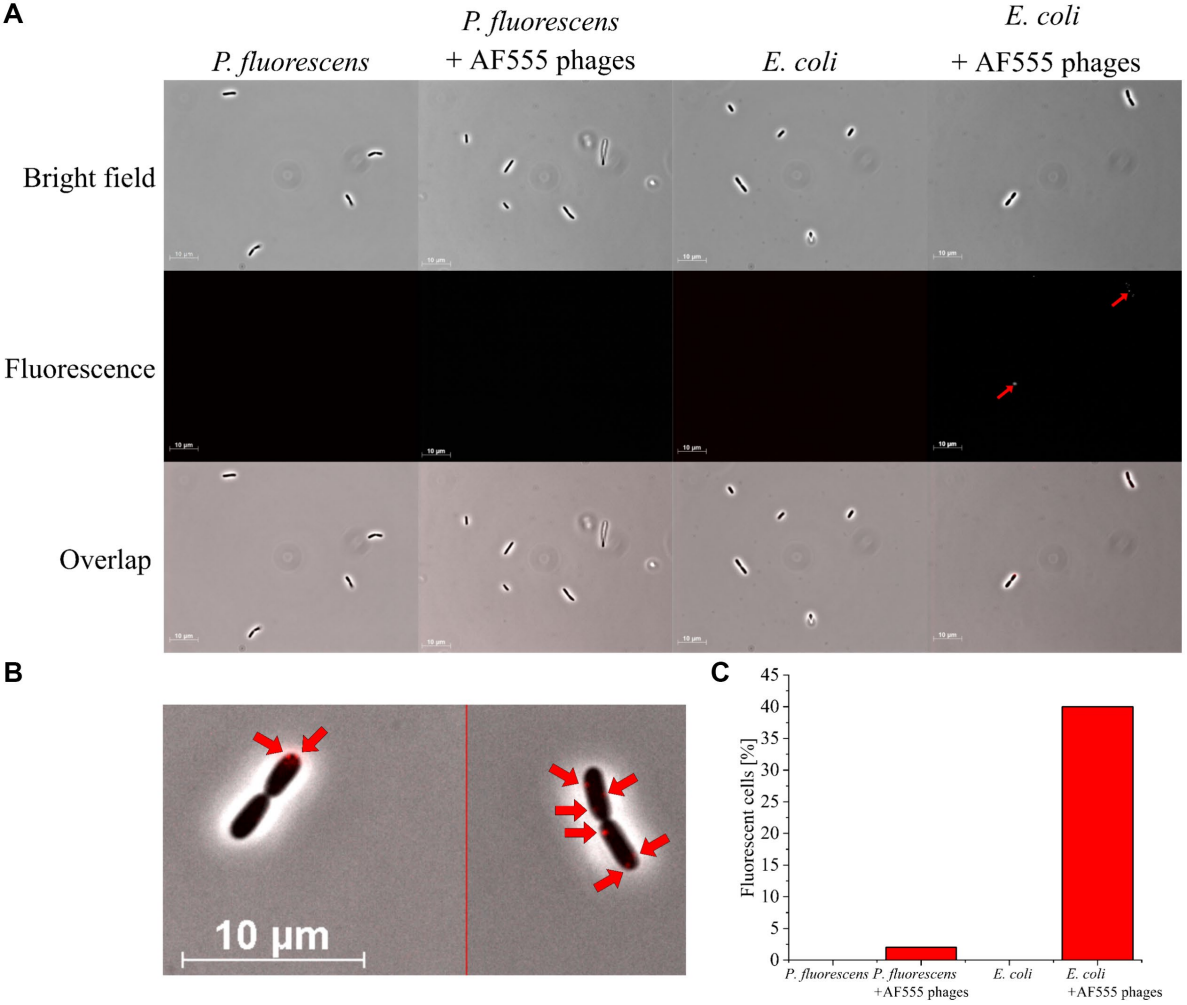
Fluorescence microscopy of *E. coli* and *P. fluorescens* (control) with AF555 phages. Cells were incubated for 30 min with and without AF555 phages. All pictures were taken with an Imager M1 fluorescence microscope (Carl Zeiss, Jena) using the software AxioVision (Version 4.8.2 SP3); brightfield and fluorescence (excitation 546/12 nm; beam splitter: FT 560; emission 575–640 nm), 1,000x, phase contrast. **(A)** Representative pictures of *P. fluorescens* and *E. coli* cells with and without AF555 phages after incubation for 30 min; only *E. coli* cells show a positive fluorescence after the addition of AF555 phages (red arrow). **(B)** *E. coli* cells with attached AF555 phages (red arrow, enlarged from overlap in **(A)**. **(C)** Percentage of fluorescent cells after scanning in greyscale mode (see Supplementary Figure S2).

Neither *P. fluorescens* nor *E. coli* showed background fluorescence at the selected wavelength (Figure 4A). *E. coli* incubated with AF555 phages emitted fluorescence (Figure 4A), whereas *P. fluorescens* incubated with AF555 phages in most cases did not emit fluorescence. Despite their small size, AF555 phages were even visible as red dots on the surface of *E. coli* cells (Figure 4B). We were even able to observe free fluorescent phages in the supernatant (Supplementary Figure S3, see Pasulka et al. (2018)).

About 40% of *E. coli* cells showed AF555 phages-specific fluorescence signals after 30 min incubation, whereas only 2% of *P. fluorescens* showed a fluorescence signal (Figure 4C). The small proportion of AF555 phages bound to *P. fluorescens* could be explained by the non-specific binding of phages to glycans of the extracellular membrane of many gram-negative bacteria (Dennehy and Abedon, 2021; Maffei et al., 2021) that are similar to carbohydrates of the *E. coli* membrane.

In summary, a workflow for AHA-labeling and CC-based addition of DBCO AF555 to phage λ proteins was established. The AF555 phages specifically bound to their host cells.

### Quantification of fluorescent phage-host complexes via flow cytometry

Fluorescence microscopy was used to monitor the absorption of AF555 phages on their host cells (Figure 4). In addition, flow cytometry was applied for automated analysis to quantify phage-host interaction (Figure 1 Step 4a).

**Figure 5:**
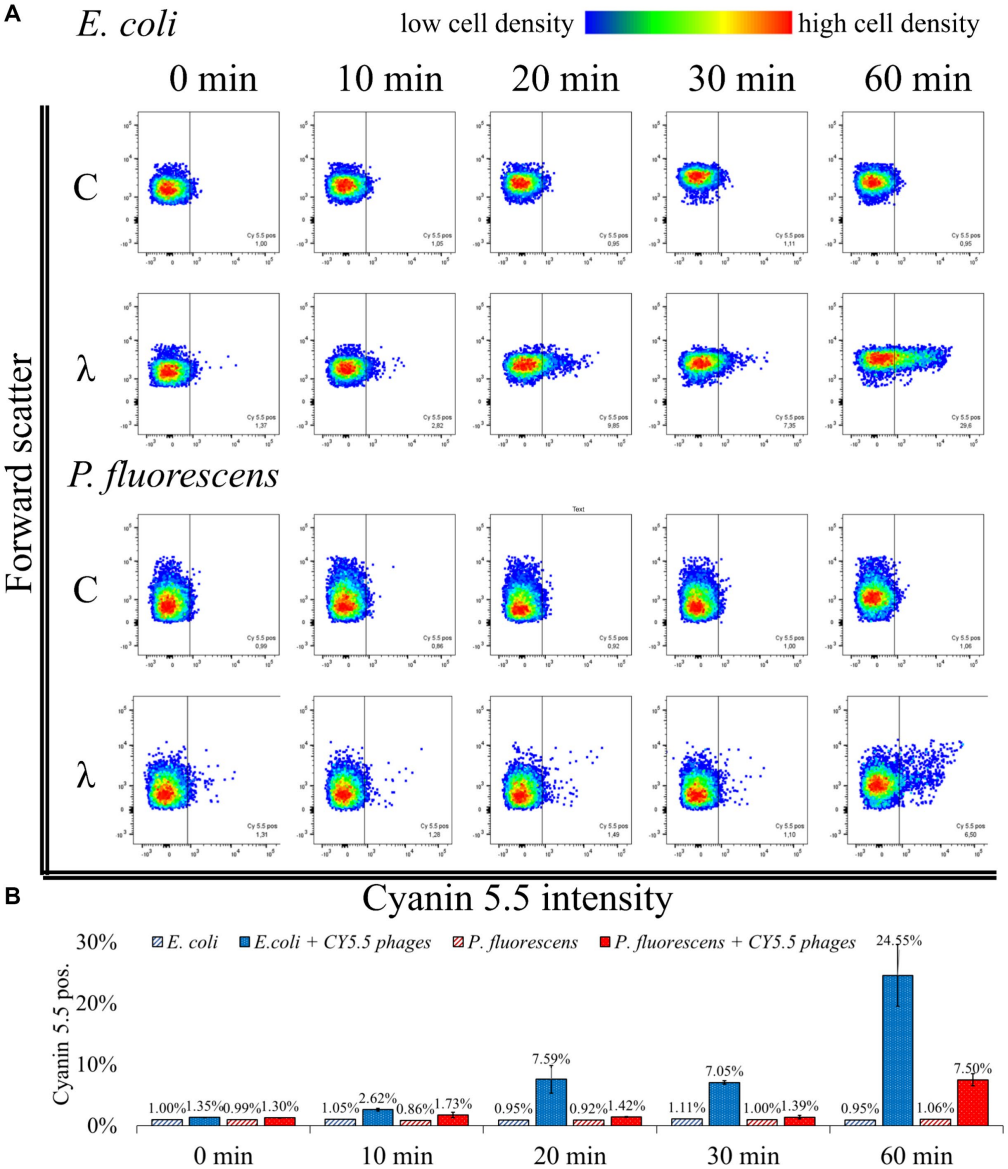
Flow cytometric analysis of phage-host complexes. **(A)** *E. coli* or *P. fluorescens* were incubated with CY5.5 phages for the indicated period (λ); control (C): without the addition of CY5.5 phages. Bacteria were harvested and treated with 4% formaldehyde for fixation. The fluorescence intensity was determined for n = 10,000 bacteria per condition. Forward scatter as an indicator of cell size was plotted against the intensity of cyanin 5.5 fluorescence. The threshold for background fluorescence of bacteria incubated without phages was set to about 1% (vertical line in the scatter plots). **(B)** Mean percentage of cyanin 5.5 positive phage-host complexes from two independent biological replicates with standard derivation. The third replicate showed different adsorption kinetics and the raw data can be found in Supplementary Table S3: FACS analysis.

First, CY5.5 phages were incubated with *E. coli* or *P. fluorescens* for up to 60 min (Figure 5A “λ”)*. E. coli* and *P. fluorescens* incubated without CY5.5 phages served as control (Figure 5A “C”). Pure bacteria (without phages) and pure CY5.5 phages were used to exclude clumped bacteria and unbound fluorescent phages from counting as positive signals for phage-host interaction analysis (see Supplementary Figure S5).

When incubated with CY5.5 phages, the percentage of fluorescent *E. coli* increased from 1.35% ± 0.04 to 24.55% ± 7.14%. A rapid increase in fluorescence of *E. coli* from 7.05% ± 0.42 to 24.55% ± 7.14% was observed between 30 min and 60 min incubation with CY5.5 phages. In contrast, the fluorescence of *P. fluorescens* did not increase significantly within the first 30 min of incubation using CY5.5 phages. After 60 min of incubation of *P. fluorescens* with CY5.5 phages, 7.50% ± 1.41% of the cells were fluorescent, indicating non-specific binding of CY5.5 phages due to the extended incubation time (Figure 5). Non-specific adsorption of CY5.5 phages to non-host cells such as *P. fluorescens* might cause false positive results. Therefore, short incubation times are suggested. Alternatively, non-specific binding could be minimized by extensive washing steps with PBS after harvest.

In our analysis of three biological replicates, we observed a slower adsorption of CY5.5 phages in one replicate, where the increase in fluorescence from *E. coli* after CY5.5 phage addition only increased from 1.12 to 4.13% after 60 min, while the fluorescence of *P. fluorescens* remained at 1.17% (Supplementary Table S3). A longer incubation time might have also increased the fluorescence signal in this experiment, similar to the other replicates.

In the case of a low number of fluorescent cells, it may be beneficial to increase the moi to enhance the number of phages bound to host cells. Since phage infections follow a Poisson distribution, a higher moi can statistically increase the number of phages per cell surface, leading to more fluorescent cells (Arkin et al., 1998; Ellis and Delbrück, 1939; Kourilsky, 1973; Marcelli et al., 2020). In particular, for unknown phage titers, the optimal adsorption conditions must be determined by analyzing different ratios of cells and phages.

In summary, the analysis of fluorescent phage-host complexes by flow cytometry confirms the results obtained from fluorescence microscopy (Figure 4) and offers the advantage of automated quantification. In addition, it allows the enrichment and isolation of fluorescent phage-host complexes by Fluorescence Activated Cell Sorting (FACS) in subsequent studies. Compared to genome staining (Deng et al., 2012), this approach has the advantage that the number of available fluorescent dyes for CC is steadily increasing, which facilitates the adaptation of the workflow to other detection methods (Van Kasteren and Rozen, 2023). In addition, only phages that are actively replicating are labeled, so that non-specifically stained fragments on cell surfaces are not analyzed. Consequently, the number of phage-host complexes identified by BONCAT and CC will be lower than in genome staining-based approaches, but the detection of those present is highly specific. Nevertheless, a combination with genome staining methods could be advantageous for subsequent studies, as the use of two dyes could allow more accurate sorting (Hardy et al., 1982).

### Purification of biotinylated phages via magnetic beads

CC of AHA-labeled proteins allows coupling of affinity tags, such as biotin, permitting specific enrichment of tagged proteins with corresponding binding partners, such as avidin (Bayer and Wilchek, 1990). Enrichment of intact biotinylated phages from complex cultures would allow subsequent analyses of the isolated phages, including DNA/RNA sequencing, LC–MS/MS-based proteomics, or follow-up infection experiments (Figure 1 Step 3b and 4b).

**Figure 6:**
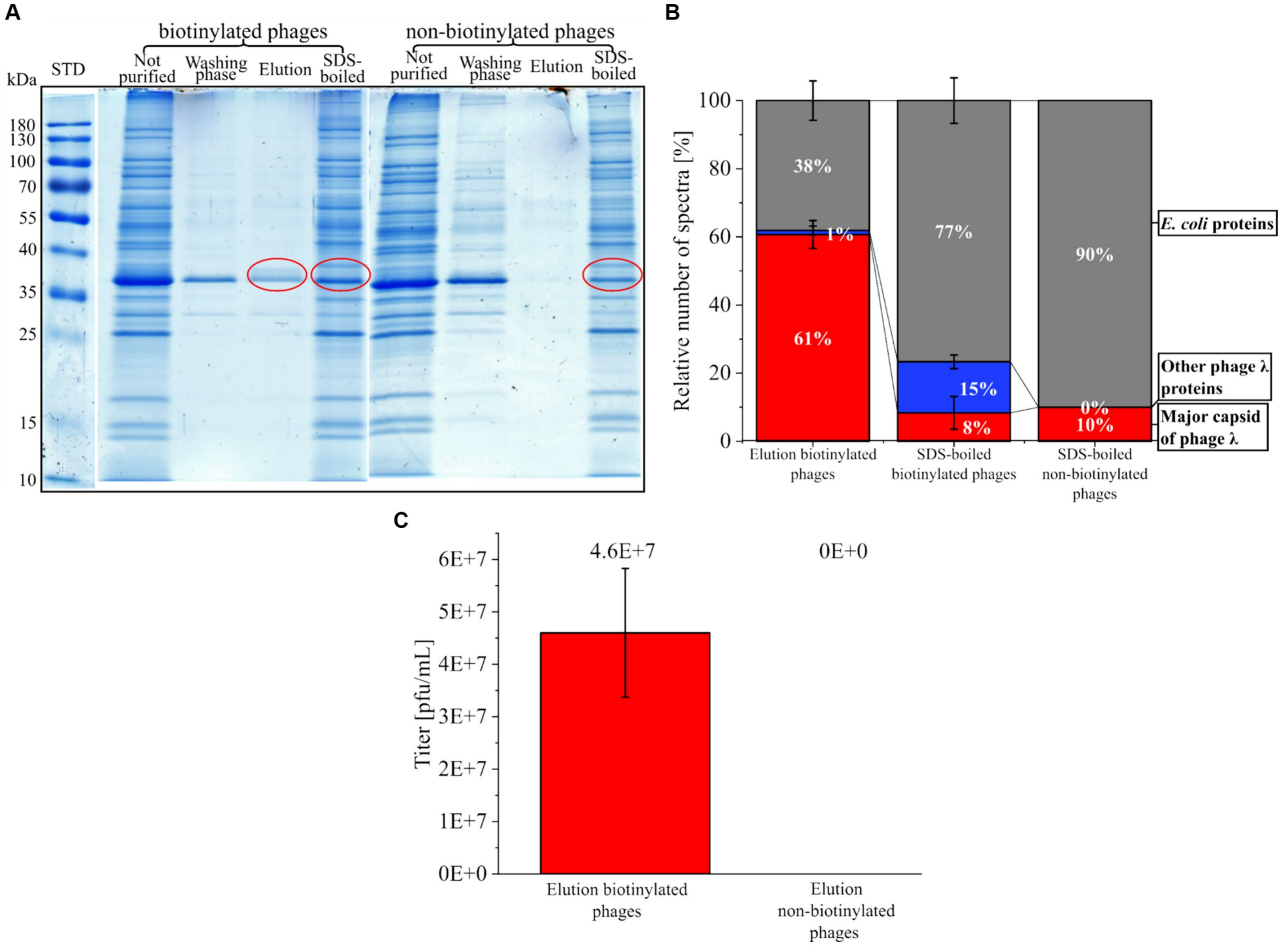
Purification of biotinylated phages with magnetic monomeric avidin beads. **(A)** SDS gel from different fractions collected during purification of phages (biotinylated or non-biotinylated) stained with Coomassie blue. The fraction “not purified” corresponds to the control (sample before the addition of beads). The “washing phase” comprises fractions of all washing steps of the beads with PBS. “Elution” includes all proteins eluted from avidin beads with a surplus of biotin. The fraction “SDS-boiled” is the collected supernatant of avidin beads after boiling (5 min, 60°C) with SDS buffer. Red circles indicate protein fractions that were further analyzed by LC–MS/MS. For the original gels, see Supplementary Figure S6. STD: protein standard (Thermo Scientific, PageRuler Prestained Protein Ladder #26616) **(B)** Relative percentage of the spectra measured with LC–MS/MS after in-gel digestion of the protein fractions marked in **A**. MS data were screened for the major capsid protein from phage λ, other phage λ proteins, and *E. coli* proteins from the background of the phage λ suspension. For raw data, see Supplementary Table S4 MS in-gel. **(C)** Plaque titer from the elution fraction of biotinylated and non-biotinylated phages; three independent experiments with mean and standard derivation below the detection limit for the elution of non-biotinylated phages.

AHA labeled phages were tagged with DBCO-PEG4-biotin (biotinylated phages) and purified with magnetic beads functionalized with monomeric avidin. The different fractions obtained were analyzed by SDS-PAGE (Figure 6A). The biotinylated phages eluted easily using a surplus of biotin (Figure 6 A “biotinylated phages” SDS gel lane “Elution”). In contrast, the elution of non-biotinylated phages (control) failed (Figure 6 A “non-biotinylated phages” SDS gel lane “Elution”). The low protein content in the collected washing fractions of beads (Figure 6 A, SDS gel lanes ‘Washing phase’) indicates that mild washing with PBS releases only low amounts of proteins from the beads with PBS. In contrast, boiling the beads with an SDS-buffer after the elution step removed many proteins from the beads, indicating non-specific binding to the bead surface, which is independent of biotinylation (Figure 6 A SDS gel lanes “SDS-boiled”). However, the non-specific binding of proteins to the monomeric avidin beads does not seem to affect the purification of phages since exclusively biotinylated phages were collected after the selective elution with biotin (Figure 6 A “biotinylated phages” SDS gel lane “Elution”).

The most abundant protein from the “Elution” of the biotinylated phages had a molecular weight of about 37 kDa. It corresponded to the major band of AF555 and CY5.5 phages identified by SDS-PAGE (Figure 6A and Supplementary Figures S1, S4). LC–MS/MS confirmed that this band mostly contained the major capsid protein of phage λ (Figure 6B red). The analysis of the SDS-boiled fractions showed a low proportion of major capsid protein but a high proportion of *E. coli* PSMs. Obviously, destroying the beads with SDS mostly released background proteins (Figure 6B), whereas a mild elution with 2 mM biotin is highly specific. Blocking the beads with amino acids or gelatin could be considered in future applications to prevent the non-specific binding of host proteins (Nicora et al., 2013; Richter et al., 2021).

Plaque assays were performed to assess the infectivity of the collected phages. Here, biotinylated phages eluted from beads showed infectivity with 4.60E+07 pfu/mL ± 1.23E+07 pfu/mL (Figure 6C), whereas the elution fraction of beads loaded with unlabeled phages showed no plaques. The missing plaques in the control experiment confirmed the specific binding of biotinylated phages, as already concluded from LC–MS/MS data.

In summary, it is possible to tag phages with biotin using CC without compromising their infectivity. Biotin tagging allows the purification of the biotinylated phages with monomeric avidin beads. The specific bead-bound phages could also be used for specific host screening in follow-up studies, where the phages bind the hosts, and the phage-host complexes are specifically released from the beads by an excess of biotin (mild condition).

## Future application of the established workflow in microbial ecology and personalized medicine

The new workflow established fluorescence labeling of phage λ for subsequent monitoring by fluorescence microscopy and flow cytometry in pure culture. A specific enrichment of infectious biotin-labeled phage λ fractions is possible using monomeric avidin beads. Other phage-host systems could be tested for future applications in microbial ecology and personalized medicine. As BONCAT approaches have been applied to a wide range of species (e.g., Babin et al., 2017; Franco et al., 2018; Metcalfe et al., 2021; Pasulka et al., 2018) no major difficulties in the transfer to other phage-host systems are anticipated. Fluorescent labeling of defined phages from phage collections would enable high throughput flow cytometry screening for alternative hosts in bacterial strain collections or the personalized selection of candidates for phage therapy using a pathogenic isolate from patient as target. In the future the screening could also be widened to non-cultivable bacteria enriched from environmental samples. Flow cytometry-based cell sorting and subsequent sequencing or proteomics could support the identification and description of new hosts for phages already available in phage collections. Induction of phage replication by MMC or other environmental stressors followed by BONCAT could also be applied to complex microbial communities (e.g., Howard-Varona et al., 2017; Jiang and Paul, 1998; Rossi et al., 2022). Phages could be separated from cells by filtration or ultracentrifugation for subsequent labeling with fluorescent dyes or affinity tags. Afterwards, flow cytometry and cell sorting could be applied to identify and characterize the corresponding hosts, including non-cultivable bacteria from the same microbial community. A preliminary experiment conducted on a co-culture of *E. coli* and *Priestia megaterium* revealed that following the addition of fluorescent phages, only the host *E. coli* exhibited fluorescence due to the specific phage adsorption (see Supplementary Figure S7).

Alternatively, biotinylated phages previously immobilized on monomeric avidin magnetic beads can be enriched from corresponding hosts for subsequent sequencing and characterization by adsorption on the surface of host cells. While applying the workflow presented here to complex microbiomes is ambitious, it holds great potential for identifying and monitoring phage-host interactions in natural environments. Comprehensive experiments are required to evaluate its application in microbiome research, a topic that extends beyond the current focus on pure cultures.

## Conclusion

A workflow for the analysis of phage λ infection in *E. coli* and the detection and purification of fluorescent phage-host complexes was established. First, phages were labeled with AHA using BONCAT. Second, labeled phages were tagged with either fluorescent dyes or biotin using CC. Using BONCAT followed by CC, is a novel strategy and flexible tool for studying microbial communities (Hatzenpichler et al., 2020). The method was demonstrated on pure cultures. Its potential application in microbial communities, including environmental or patient samples, requires further assessment of the workflow under complex environmental conditions. Furthermore, fluorescent phages could be applied for specific screening of phage libraries for the therapy of infectious diseases. Finally, due to the increasing repertoire of substances for CC, it can be safely assumed that the BONCAT and CC approach will evolve into a very flexible toolbox for phage research and therapy (van Kasteren and Rozen, 2023).

## Data availability statement

The datasets presented in this study can be found in online repositories. The names of the repository/repositories and accession number(s) can be found at: https://www.ebi.ac.uk/pride/archive/, PXD044316.
